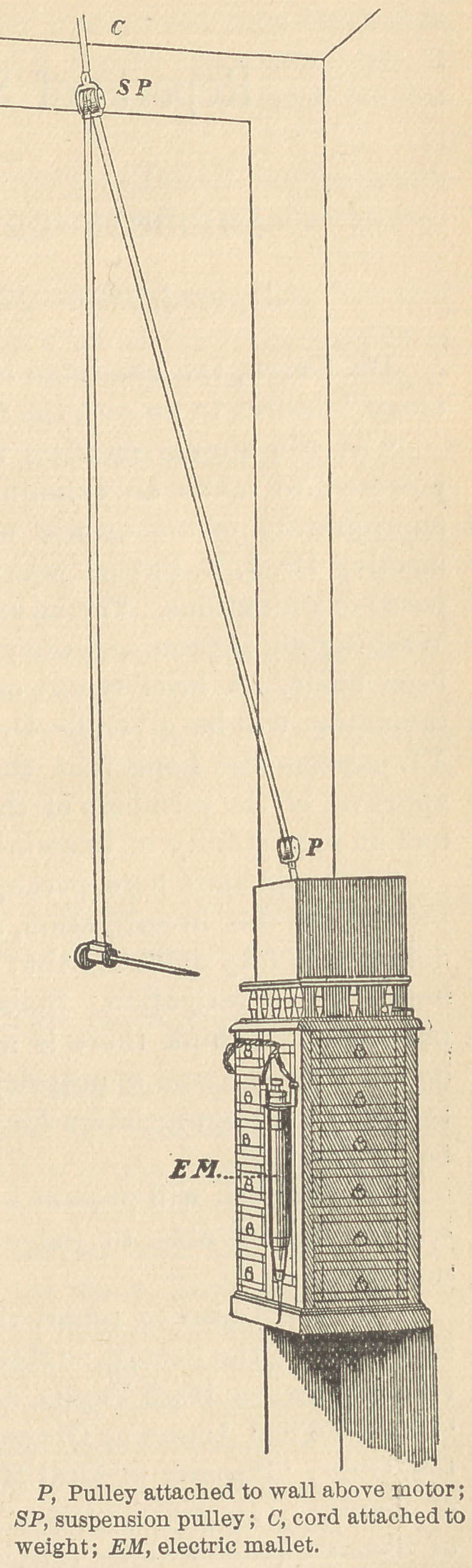# A New Method of Running the Suspension Dental-Engine by the Electric Current

**Published:** 1891-11

**Authors:** T. A. Fletcher

**Affiliations:** New York


### A NEW METHOD OF RUNNING THE SUSPENSION DENTAL-ENGINE BY THE ELECTRIC CURRENT.

BY T. A. FLETCHER, D.D.S., NEW YORK.

I HAVE applied to my suspension engine the Edison current on 110-volt circuit. The motor is placed on the top of my instrument cabinet, covered with an ebony case to match the cabinet. The shaft of the motor passes out on the wall side, with the pulley out of sight, and, being noiseless, attracts no attention. It runs with perfect steadiness. Just above the cabinet a set of pulleys is attached, over which the cord passes to a pulley suspended and governed by a cord and weight. This cord passes across the ceiling to the corner of the room. The cord passing through the suspended pulley is continued to the hand-piece. It will be observed by reference to the illustration that the hand-piece can be run at any angle and from either side of the chair.

The resistance-box is in the corner of the room. Double resistance is used,—the lower to govern the speed of the engine, and the upper for mouth-lamp and mallet.

The mallet best adapted for this system is the Gibbs electric mallet, represented in front of the cabinet.

The satisfaction which I have derived from this arrangement has led to the conclusion that it is the best way yet devised for the application of the electric current to the dental-engine.

The mouth-lamp I use is of the Edison approved style. To this I have adapted a black rubber hood, and set the lamp in place with plaster, which serves the double purpose of a reflector and cutting off the heat, protecting the tissues for an indefinite period.

The starting and instantaneous device, also the reversal of rotation of my motor, are controlled by a simple pedal connection that can be placed under the chair.

During the time I have had this in use, it has not only given me entire satisfaction, but my patients express themselves more than pleased with the change.

Credit is due Mr. George M. Wheeler, my electrician, who has furnished me with his noiseless reversible motor and magnetic stopping attachment.

*P*, Pulley attached to wall above motor; *SP*, suspension pulley; *C*, cord attached to weight; *EM*, electric mallet.

The workings of this arrangement can probably be understood by the illustration; but I will be pleased to exhibit it at my office, 67 West Fifty-fourth Street, New York City.

## Figures and Tables

**Figure f1:**